# Prevalence and Clinical Impacts of Canine Herpesvirus‐1 (CHV‐1) in Dogs: A Review of Reproductive Effects and Ocular Lesions

**DOI:** 10.1002/vms3.70682

**Published:** 2025-11-19

**Authors:** Sina Soleimani, Mohammadreza Ghorani, Amir Mohammad Naghshe Javaheri, Mahdiye Shirafkan, Hadi Bakhtiari

**Affiliations:** ^1^ Razi Vaccine & Serum Research Institute Agricultural Research Education and Extension Organization (AREEO) Karaj Iran; ^2^ Department of Pathobiology Faculty of Veterinary Medicine University of Tabriz Tabriz Iran; ^3^ Doctor of Veterinary Medicine, Faculty of Veterinary Medicine University of Tabriz Tabriz Iran; ^4^ Department of Laboratory Medicine Ar.C., Islamic Azad University Arak Iran; ^5^ Applied Neuroscience Research Center Ar.C., Islamic Azad University Arak Iran

**Keywords:** canine herpesvirus‐1 (CHV‐1), latent infection, ocular lesions, prevalence, reproductive effects

## Abstract

Canine herpesvirus‐1 (CHV‐1), as a member of the *Varicellovirus*, subfamily Alphaherpesvirinae and family Herpesviridae, is mainly transmitted at birth but can also spread venereally and transplacentally. In addition, CHV‐1 establishes a latent carrier state in the body and can reactivate due to stress or immunosuppression. CHV‐1 distribution varies worldwide but is believed to have a global distribution. CHV‐1 infection in adult canines can manifest as a spectrum of ocular from eyelid inflammation (blepharitis) and conjunctival inflammation (conjunctivitis) to more severe corneal conditions, including ulcerative and non‐ulcerative keratitis. Moreover, CHV‐1 in adult canines can lead to a range of reproductive effects, from submucosal vascular congestion and bleeding to foetal expulsion and preterm birth of live offspring. Subclinical or mildly symptomatic upper respiratory tract disease can manifest in juvenile and adult canines. Prophylactic topical antimicrobial therapy is recommended to prevent disease progression in dogs with CHV‐1 ocular disease. However, the environmental temperature increase for affected puppies fails to modify the disease progression. Environmental variables, including breeding facility size and animal population density, facilitate herpesvirus transmission and subsequent immune responses. There are various diagnostic techniques, but the most prevalent method is polymerase chain reaction (PCR) for viral DNA detection. Due to the global distribution of the virus and its effects, such as ocular and reproductive effects and subsequent financial losses, it is recommended that infected dogs be identified and treated promptly, as well as prevent its transmission.

## Graphical abstract

**Taxonomy**: Herpesviridae, Alphaherpesvirinae and *Varicellovirus*.

**Diagnosis**: PCR for genetic detection.

**Transmission**: Birth, venereal and transplacental.

**Pathogenesis**: Ocular lesions; blepharitis, conjunctivitis and ulcerative and non‐ulcerative keratitis.

Reproductive effects: submucosal vascular congestion and bleeding to foetal expulsion and preterm birth of live offspring.

Subclinical or mildly symptomatic URTD.

Latency and reactivation (stress/immunosuppression).

**Management**: Prophylactic topical antimicrobial therapy in dogs with CHV‐1 ocular disease.

Enhance breeding facility size and animal population density.

Identify and treat infected dogs promptly in order to prevent its transmission.

Antimicrobial therapy and antiviral agents.

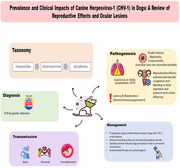

## 1 Introduction

Canine herpesvirus type 1 (CHV‐1) is taxonomically classified as a member of the genus *Varicellovirus* within the subfamily Alphaherpesvirinae of the family Herpesviridae. The etiological agent, subsequently classified as CHV‐1, was initially isolated and characterized in 1965 by three independent research groups through the examination of fatal disease cases in neonatal canines and primary cultures derived from canine renal tissue (Carmichael et al. 1965; Spertzel et al. 1965; Stewart et al. 1965).

CHV‐1 infection manifests as a severe, systemic, necrotizing and haemorrhagic disease with a fatal outcome in susceptible neonatal canines. However, in post‐neonatal individuals, the infection often proceeds subclinically (Carmichael et al. 1965; Evermann et al. 2011). CHV‐1 is implicated in the pathogenesis of reproductive disorders in adult canines, including embryonic loss through mechanisms such as resorption, abortion and foetal demise.

CHV‐1 infection can induce the formation of vesicular eruptions on the mucosal surfaces of the vestibule and vagina in female canines, often accompanied by acute vaginitis. Similarly, males may exhibit vesicular lesions on the penis and prepuce. CHV‐1 has been sporadically linked to the development of upper respiratory tract and ocular pathologies in adult canines (Ledbetter 2013).

Primary infection in susceptible neonatal canines predominantly occurs through oronasal exposure to the virus during the parturition process, acquired from the maternal birth canal. Additional modes of transmission for CHV‐1 include both venereal and transplacental routes (Evermann et al. 2011). Following either an acute clinical presentation or a subclinical infection, canines transition into a latent viral carrier state. In this phase, CHV‐1 can be detected within the trigeminal and lumbosacral ganglia, salivary glands, tonsils and hepatic tissue, despite the absence of overt clinical manifestations (Burr et al. 1996).

Reactivation of latent CHV‐1 can be precipitated by physiological stressors or experimentally induced immunosuppression through the administration of immunosuppressive pharmacotherapeutics or anti‐lymphocyte antibodies (Ledbetter et al. 2012). CHV‐1 is characterized by weak immunogenicity, resulting in a variable and transient humoral immune response. Neutralizing antibody titres exhibit fluctuations within a 4–8 week period post‐exposure, with a limited persistence typically lasting only a few months (Greene and Carmichael 2006; Nöthling et al. 2008).

A multitude of epidemiological factors can influence the seroprevalence of CHV‐1 within a given population. CHV‐1 seroprevalence is significantly higher within large‐scale and breeding kennel environments with a documented history of neonatal infections. Epidemiological studies have demonstrated a positive correlation between increasing age, mating status in males and advanced reproductive age in females with elevated anti‐CHV‐1 antibody titres (Ronsse et al. 2004).

CHV‐1 is believed to have a global distribution. CHV‐1 has been documented in numerous countries globally. Although its prevalence is ubiquitous, seroprevalence studies have revealed significant geographical disparities in antibody titres among canine populations. Heterogeneity in serological methodologies and sampling populations contributes significantly to the observed variability in CHV‐1 seroprevalence across geographic regions. Discrepancies in diagnostic techniques and the specific demographics of study populations can substantially impact the estimation of CHV‐1 antibody prevalence.

This review surveys CHV‐1 and its risk factors, prevalence, diagnosis and treatment. This review aims to summarize the current knowledge regarding the prevalence, clinical implications and management of CHV‐1 infections, with a focus on reproductive and ocular effects. The necessity of this review stems from several critical factors. First, the virus's pathogenesis, characterized by latency, reactivation under stress and transmission through bodily fluids, complicates disease management in breeding kennels and multi‐dog households. Second, variable clinical presentations (e.g., asymptomatic carriers, acute fatal haemorrhagic disease or reproductive failure in adults) hinder timely diagnosis, often resulting in overlooked outbreaks. Furthermore, recent advances in molecular virology, including genomic studies and vaccine development, have yet to be synthesized into a cohesive resource for researchers and clinicians.

## 2 Materials and Methods

### 2.1 Literature Search Strategy

A systematic literature search was conducted to identify all relevant publications on CHV‐1. All of the electronic databases, including PubMed, Scopus and Web of Science, were used. Search terms included the following: ‘CHV‐1’, ‘CHV‐1’ and ‘canid herpesvirus’, ‘reproductive failure dogs’, ‘canine abortion’, ‘ocular keratitis dog’, ‘canine neonatal mortality’ and ‘CHV‐1 prevalence’. Reference lists of retrieved articles were also hand‐searched for additional relevant studies.

### 2.2 Study Selection and Eligibility Criteria

Studies were included if they were original research articles, case reports or reviews published in English that focused on CHV‐1's virology, pathogenesis, prevalence, clinical manifestations (reproductive or ocular), diagnosis or treatment. Studies on other canine viruses without specific CHV‐1 data were excluded in this study.

### 2.3 Data Extraction and Quality Assessment

Data extracted from included studies encompassed author, year, country, study design, population characteristics (e.g., kennel vs. pet dogs), sample size, diagnostic methods used (such as polymerase chain reaction [PCR], serology and virus isolation), key findings on prevalence and clinical outcomes. The quality of included studies was appraised on the basis of sample size, clarity of methodology, appropriateness of diagnostic techniques and statistical analysis. The heterogeneity of diagnostic methods and study populations is acknowledged as a significant factor influencing the interpretation of pooled results.

## 3 Ocular Lesions

CHV‐1 has been established as a primary etiological agent in severe systemic neonatal infections and reproductive pathology, including infertility and abortion, for several decades. Although the association between CHV‐1 and these conditions is well‐documented, the ocular manifestations of the virus in adult canines have only recently been definitively characterized.

CHV‐1 is best known for its devastating effects on neonatal puppies, but its role in ocular disease particularly in adult dogs has been increasingly documented. Ocular lesions are often underdiagnosed due to nonspecific symptoms and viral latency, but emerging studies emphasize their clinical significance. CHV‐1 was identified as a cause of chronic, refractory corneal ulcers and stromal keratitis in two adult dogs.

Clinically, CHV‐1 infection in adult canines can manifest as a spectrum of ocular lesions and adnexal pathologies. These can range from eyelid inflammation (blepharitis) and conjunctival inflammation (conjunctivitis) to more severe corneal conditions, including ulcerative and non‐ulcerative keratitis. Ocular and adnexal pathologies associated with CHV‐1 can arise during both initial and subsequent infections (Evermann et al. 2011; Ledbetter 2013).

Both individually owned domestic dogs and dogs maintained in communal environments, such as research facilities or kennels, are susceptible to CHV‐1 infection. Initial ocular infections caused by CHV‐1 typically manifest bilaterally. However, the severity and specific clinical features of the disease can exhibit asymmetrical presentation between the affected eyes of individual dogs. Subsequent ocular infections caused by CHV‐1 can manifest as either unilateral or bilateral ocular disease.

The clinical presentation of ocular CHV‐1 infections, both initial and recurrent, is modulated, at least in part, by the age and immunological status of the affected canine. In healthy, immunocompetent adult dogs, primary CHV‐1 ocular infections typically manifest as subclinical or mild ocular disease that resolves spontaneously without intervention. Ocular manifestations of CHV‐1 infection are often exacerbated in immunosuppressed adult dogs, characterized by increased severity and prolonged disease duration (Ledbetter 2013).

Ocular manifestations of CHV‐1 infection in adult dogs exhibit a diverse clinical spectrum, encompassing a range of signs and lesions (Ledbetter et al. 2006; Ledbetter et al. 2009; Ledbetter et al. 2009; Malone et al. 2010; Gervais et al. 2012). Unlike the systemic and often fatal ocular manifestations observed in foetal and neonatal canines, CHV‐1 infections in adult dogs primarily affect the ocular surface and adnexal structures. The clinical presentation of CHV‐1 ocular disease in adult dogs is heterogeneous, often characterized by a combination of blepharitis, conjunctivitis, ulcerative keratitis and non‐ulcerative keratitis (Ledbetter 2013).

Common clinical signs associated with CHV‐1 ocular infection include blepharospasm, photophobia, ocular rubbing, elevation of the third eyelid and ocular discharge (Ledbetter et al. 2006; Ledbetter et al. 2009; Ledbetter et al. 2009; Gervais et al. 2012). CHV‐1‐induced blepharitis is characterized by eyelid inflammation manifested as redness, swelling, exudate formation, crusting, ulceration and hair loss. Conjunctivitis is the most prevalent ocular manifestation of CHV‐1 infection in adult canines, often presenting as an isolated lesion but frequently co‐occurring with other eyelid or corneal pathologies (Ledbetter et al. 2009; Ledbetter et al. 2009; Malone et al. 2010).

Ulcerative keratitis is a commonly observed corneal lesion associated with CHV‐1 ocular disease in adult canines (Ledbetter et al. 2006; Ledbetter et al. 2009; Gervais et al. 2012). The detection and quantification of ocular viral shedding during both primary and recurrent CHV‐1 infections is crucial for understanding the epidemiology of the disease and for confirming a clinical diagnosis. This process involves the release of infectious viral particles from infected ocular tissues, facilitating transmission to susceptible canine hosts (Ledbetter 2013).

### 3.1 Ocular Lesions Diagnosis and Treatment

A definitive diagnosis of active ocular CHV‐1 infection can be established through the analysis of corneal or conjunctival samples using various diagnostic techniques. These include PCR for viral DNA detection (viral DNA was detected via PCR in corneal tissues), virus isolation through cell culture, electron microscopy for direct viral visualization (histopathology revealed intranuclear inclusion bodies) or immunofluorescent antibody assay for antigen detection (Evermann et al. 2011; Ledbetter et al. 2006; Schulze and Baumgärtner 1998; Decaro et al. 2010).

Prophylactic topical ocular antimicrobial therapy is recommended for all dogs with CHV‐1 ocular disease to prevent secondary bacterial infections, which can exacerbate disease progression, leading to severe ocular sequelae, including corneal perforation. Topical ocular atropine administered to induce mydriasis (pupillary dilation) can provide symptomatic relief in dogs with corneal disease by reducing ocular discomfort.

Clinical reports have documented the apparent efficacy of topical antiviral therapy in managing CHV‐1 ocular infections in dogs. Antiviral agents, such as idoxuridine 0.1% ophthalmic solution, trifluridine 1% ophthalmic solution and cidofovir 0.5% ophthalmic solution, have been reported to produce positive outcomes in some cases (Ledbetter et al. 2009; Malone et al. 2010; Gervais et al. 2012).

### 3.2 Differential Diagnosis of Ocular CHV‐1

The ocular manifestations of CHV‐1 are nonspecific and can closely mimic those of other important ocular pathogens. Canine adenovirus‐1, though rare due to vaccination, can cause anterior uveitis and keratitis (blue eye). Canine distemper virus can cause optic neuritis and chorioretinitis. The most common and clinically significant differential is Feline Herpesvirus‐1, which is a primary cause of ulcerative keratitis in cats and has been documented in dogs. However, CHV‐1 is uniquely associated with bilateral conjunctivitis and keratitis in the context of a kennel outbreak or in conjunction with reproductive failure. Bacterial keratitis (e.g., *Staphylococcus*, *Pseudomonas*), fungal keratitis and keratoconjunctivitis sicca (dry eye) must also be ruled out through thorough ophthalmic examination, Schirmer tear testing and corneal cytology/culture (Ledbetter et al. 2009; Decaro et al. 2010).

## 4 Reproductive Effects

CHV‐1 is a cytoadherence pathogen disseminated via either cellular coalescence or passive vector‐mediated dispersal. Following epithelial progeny production at the inoculation site, leukocyte‐associated viraemia dissemination ensues, resulting in tropism for a broad spectrum of neural and lymphoid tissues. Disseminated maternal infection during gestation can induce placental inflammation, facilitating transplacental viral transfer to the offspring (Hashimoto et al. 1982).

Irrespective of the inoculation route, all canines exhibit nasal mucosal viral shedding during the acute phase of infection (Miyoshi et al. 1999). Simultaneous viral excretion and concomitant genital and respiratory tract infections can manifest despite the presence of circulating humoral immune factors. Horizontal transmission from infected males to susceptible females via venereal contact is considered an insignificant mode of viral propagation. Conversely, genital viral localization in females represents a primary conduit for perinatal transmission to offspring (Greene 2005).

Within the adult host population, infection is prevalent without overt clinical manifestations. However, during the initial acute phase, mucosal viral entry can induce localized inflammatory responses characterized by lymphoid follicular hyperplasia, submucosal bleeding and vascular congestion (Evermann et al. 2011; Ronsse et al. 2004; Morresey 2004). Subclinical or mildly symptomatic upper respiratory tract disease can manifest in juvenile and adult canines. Epithelial vesicle formation has been observed to coincide with the initiation of proestrus in females, subsequently undergoing spontaneous resolution during the anestrous phase (Greene 2005).

The gestational timing of CHV‐1 inoculation in nulliparous pregnant canines can lead to a spectrum of foetal outcomes, including subclinical foetal loss, foetal desiccation, foetal expulsion or preterm birth of live offspring. Canine dams with a history of CHV‐1 exposure and subsequent seroconversion typically have uncomplicated deliveries. Passive transfer of maternal humoral or cellular immune components via colostrum from seropositive dams confers protective immunity in puppies against the clinical manifestations of CHV‐1 infection (Greene 2005; Kraft et al. 1986).

The temporal window of peak susceptibility to CHV‐1‐induced pathology has been precisely defined as 6 weeks, encompassing 3 weeks antepartum and 3 weeks postpartum (Evermann 1989). Postnatal viral exposure is more prevalent. In litters exposed peripartum or postnatally, clinical disease primarily manifests between the first and third weeks of life. The interval between viral exposure and disease onset, or incubation period, spans 3–7 days. Clinical presentation is nonspecific, characterized by rapid, shallow respiration, abdominal discomfort, anorexia and emesis (Greene 2005).

### 4.1 Reproductive Effects Diagnosis and Treatment

Serological assays have historically been employed for CHV‐1 diagnosis; however, the virus's limited immunogenicity can compromise the accuracy of such diagnostic approaches. Following CHV‐1 exposure and potential abortion in canines, antibody titres exhibit a rapid and transient elevation, typically peaking and declining within a 4–8‐week period, with an estimated duration of no more than 60 days post‐exposure (Evermann 1989). Necrotic tissue regions containing characteristic intracellular viral inclusions are pathognomonic of CHV‐1 infection and are disseminated throughout multiple organ systems (Hashimoto et al. 1979).

Therapeutic interventions for CHV‐1‐infected puppies are typically ineffective once clinical signs manifest due to the rapidly progressive and severe nature of the disease. Augmentation of environmental temperature for affected individual puppies fails to modify the disease progression. Case fatality rates within outbreak settings may be mitigated through intraperitoneal administration of serum derived from previously infected bitches. A singular dosage ranging from 1 to 2 mL is deemed adequate (Greene 2005).

### 4.2 Differential Diagnosis of Reproductive CHV‐1

Reproductive failure in dogs has multiple infectious and non‐infectious causes. CHV‐1‐induced abortion typically results in puppy autolysis and is often late‐term. Key infectious differentials include the following:

- ***Brucella canis***: The classic zoonotic cause of late‐term abortion, infertility and orchitis. Serological testing is mandatory for any dog with reproductive failure.
- **Canine brucellosis**: Abortion, stillbirths and infertility.
- **Other pathogens**: *Escherichia coli*, *Streptococcus* spp., *Toxoplasma gondii* and *Neospora caninum* can also cause abortion and should be considered based on histopathology and PCR.
  Non‐infectious causes include hormonal imbalances, genetic defects and nutritional deficiencies. The lack of pathognomonic signs makes definitive diagnosis reliant on laboratory confirmation (PCR, histopathology with intranuclear inclusion bodies) from the foetus or placenta (Ledbetter et al. 2006).

## 5 Risk Factors

CHV‐1 is a highly contagious pathogen whose transmission and severity are shaped by host susceptibility, environmental conditions and management practices. In vitro investigations have been conducted to characterize CHV‐1 and elucidate its pathogenic mechanisms (Hashimoto et al. 1982). In vitro studies have been conducted to elucidate CHV‐1 characteristics and pathogenesis, including viral latency and the potential for reactivation following prednisolone administration (Okuda et al. 1993; Okuda et al. 1993).

S. Il‐bok isni et al. (1994) and S. V. Gucht et al. (2001) independently observed a positive correlation between canine age and seroprevalence, with a statistically significant increase in antibody titres detected in dogs older than 6 and 12 months, respectively. Lacheretz and Cognard (1998) documented a progressive acquisition of antibodies in dogs up to 6 years of age, with a markedly lower antibody prevalence of 0.7% observed in the under‐2‐year‐old cohort.

S. Il‐bok isni et al. (1994) reported a modest gender disparity in CHV‐1 seroprevalence, with males exhibiting a higher antibody positivity rate (42%) compared to females (33%). In contrast to other species with established evidence of herpesvirus recurrence during gestation and parturition (Bujko et al. 1988; Thiry et al. 1985), the influence of sex steroids on CHV‐1 reactivation and subsequent seroconversion in canines remains largely unexplored. Clinical manifestations of CHV‐1 reactivation have been documented with increased frequency in female dogs during oestrus and parturition (Anvik 1991).

Historically, CHV‐1 has been frequently isolated as an etiological agent in canine infectious respiratory disease complex cases. However, its role as a primary pathogen in the respiratory tract is considered to be of secondary importance (Appel et al. 1969). Environmental variables, including breeding facility dimensions and animal population density, have been shown to facilitate the transmission of herpesvirus and the development of subsequent immune responses (Leontides et al. 1994; Maes et al. 2000; McDermott et al. 1997).

In total, the existing risk factors are discussed as follows:

Overcrowding: Large kennels or multi‐dog households with high dog density facilitate viral spread through direct contact (nasal secretions, saliva) and aerosols (Krogenæs et al. 2014).
Fomite transmission: CHV‐1 survives for 24–48 h on surfaces (food bowls, bedding) at room temperature. Poor disinfection protocols enable indirect transmission (Evermann et al. 2011).
Neonates (<3 weeks old): Lack maternal antibody protection (if the dam is seronegative) and thermoregulatory capacity. Mortality approaches 100% due to systemic necrotizing vasculitis (Buonavoglia et al. 2001).
Stress‐induced reactivation: Shipping, rehoming or prolonged confinement in vehicles elevates cortisol levels, triggering viral shedding in latently infected carriers (Ledbetter 2013).
Introduction of new dogs: Seronegative dams or stud dogs entering a CHV‐1‐endemic kennel risk acute infection during pregnancy, leading to abortion storms (Ronsse et al. 2005).

## 6 Prevalence and Epidemiological Challenges

Reported seroprevalence rates for CHV‐1 exhibit extreme geographical variability, ranging from 20% to over 85% in canine populations (Nöthling et al. 2008) (Krogenæs et al. 2014). This wide disparity is not necessarily indicative of true differences in viral distribution but rather highlights critical methodological challenges inherent in CHV‐1 research.

A. Krogenæs et al. (2014) found CHV‐1 infection prevalent among breeding bitches in eastern Norway. However, traditional risk factors showed no association, seasonal variations, prior litters and participation accounted for a substantial portion (67%–78%) of antibody titre differences.

Serological studies in domestic dogs have yielded highly variable prevalence rates, ranging from zero to complete seropositivity across different geographical regions (Sykes and Greene 2012). Ronsse et al. (2002) determined a 45.75% prevalence of antibodies against CHV‐1 within the Belgian canine population. A subsequent study conducted in 2008 within the Gauteng Province of South Africa revealed a 22% seroprevalence of CHV‐1 antibodies among canine populations, as determined by both serum neutralization test (SNT) and enzyme‐linked immunosorbent assay (ELISA) methodologies (Nöthling et al. 2008). A separate study conducted by Dahlbom et al. (2009) revealed a significantly higher CHV‐1 seroprevalence of 81.5% within the Finnish canine population.

A high seroprevalence rate of 85.5% for CHV‐1 infection was established within the Norwegian canine population through the application of immunoperoxidase monolayer assay (IPMA) (Krogenæs et al. 2014). A concurrent study conducted in Turkey employed both virus neutralization test (VNT) and ELISA to assess CHV‐1 seroprevalence. ELISA demonstrated a higher antibody detection rate of 39.3% compared to the 29.4% identified through VNT (Yeşilbağ et al. 2012). A study conducted by Babaei et al. (2010) determined an overall CHV‐1 seroprevalence of 20.7% within the canine population through the application of an indirect immunofluorescence antibody (IFA) assay.

Real‐time PCR analysis detected CHV‐1 DNA in 15% (21/140) of examined canine samples (Rezaei et al. 2020). Losurdo et al., employing real‐time PCR, demonstrated that CHV‐1 infection can manifest as a fatal systemic disease with characteristic clinical and histopathological findings. Furthermore, the study revealed the potential for prolonged viral shedding via nasal, ocular and faecal routes (Losurdo et al. 2018).

Cargnelutti et al. (2015) documented a systemic fatal disease outbreak characterized by neonatal mortality. The study employed histopathology, VNT and PCR for diagnostic confirmation (Cargnelutti et al. 2015). A study by Larsen et al. (2015) in Denmark revealed a 22.8% prevalence of CHV‐1 infection among 57 deceased puppies as determined by qPCR. Histopathological and in situ hybridization findings exhibited inconsistent correlation with the PCR results (Larsen et al. 2015).

Gadsden et al. (2012) documented a fatal case of CHV‐1 infection in a 9‐year‐old spayed female Bichon Frise. The diagnosis was confirmed through PCR, immunohistochemistry and in situ hybridization (Gadsden et al. 2012). A Mexican study confirmed CHV‐1 infection in deceased puppies through a comprehensive diagnostic approach encompassing histopathology, direct immunofluorescence (IF), electron microscopy and PCR (Rezaei et al. 2020).

Pratelli et al. (2014) reported seroprevalence rates of 14.6% and 18.6% for CHV‐1 in an Italian population, as determined by SNT and in‐house IF, respectively. However, no CHV‐1 DNA was detected in any of the vaginal swab samples analysed (Pratelli et al. 2014). A separate Italian study yielded negative results for CHV‐1 antibodies in all serum samples analysed using both SNT and nested PCR assays (Bottinelli et al. 2016).

Li et al. (2016) demonstrated through experimental models that CHV‐1 exhibits superior replication and invasion capabilities within the vaginal mucosa compared to the respiratory mucosa, as evidenced by the greater extent and depth of viral antigen‐positive cell clusters. Despite the absence of statistically significant breed‐related differences reported by Yeşilbağ et al. (2012), a higher prevalence of CHV‐1 was observed in Golden Retrievers (56.2%) compared to Terriers (50%) within the Turkish canine population. Previous research has indicated that housing conditions do not significantly influence the prevalence of CHV‐1 (Ronsse et al. 2002; Babaei et al. 2010; Pratelli et al. 2014). Conversely, numerous studies have documented a higher prevalence of CHV‐1 infection within kennel or shelter environments compared to individually owned dogs (Yeşilbağ et al. 2012). Dahlbom et al. (2009) identified a correlation between elevated antibody titres and reproductive complications; however, conflicting evidence exists, with other studies demonstrating no significant correlation between CHV‐1 infection and reproductive disorders (Pratelli et al. 2014; Krogenæs et al. 2012).

Ronsse's study revealed no significant correlation between reproductive status and CHV‐1 antibody titres, despite a trend towards increased abortion rates in seropositive breeding kennels (Ronsse et al. 2004, 2005). Conversely, other research studies have failed to establish a definitive link between CHV‐1 antibody titres and reproductive outcomes (Bottinelli et al. 2016; Krogenæs et al. 2012). Ström Holst et al. (2012) postulated that the reactivation of latent CHV‐1 infection during pregnancy is unlikely under optimal sanitary and management conditions.

The prevalence of CHV‐1 infection exhibits geographical variability and is significantly influenced by factors such as sampling methodology and the specific characteristics of study populations. The diagnostic accuracy of different methodologies employed to detect CHV‐1 can vary significantly, influencing the overall seroprevalence estimates. CHV‐1 is characterized by its poor immunogenicity, resulting in a transient antibody response that typically wanes within a few months post‐infection (Ronsse et al. 2004; Bottinelli et al. 2016). Standardization of serological assays for CHV‐1 is lacking, resulting in inconsistent results across different laboratories and hindering comparative analysis of seroprevalence data. Consequently, serological methods may underestimate the true prevalence of CHV‐1 infection due to limitations in assay standardization and the transient nature of antibody responses. PCR is widely recognized as a gold standard diagnostic technique for detecting both active CHV‐1 infections in puppies and latent viral reservoirs in adult dogs (Decaro et al. 2010; Sykes and Greene 2012).

The study by Ada Rota et al. revealed a high prevalence of CHV‐1 infection within the Piedmont breeding dog population, although a notable degree of heterogeneity existed among kennels, with some exhibiting a complete absence of the virus. Within kennels where CHV‐1 infection was detected, seroprevalence rates were notably high, with a substantial proportion of dogs exhibiting elevated antibody titres. Canine females exhibit a post‐pubertal increase in antibody titres, suggesting the development of protective immunity during the reproductive period, potentially conferring benefits to both dam and offspring.

Despite the potential for naturally acquired immunity, vaccination of seronegative pregnant bitches is recommended to mitigate the risk of CHV‐1 infection in kennels with endemic viral circulation. Prophylactic vaccination is advisable for kennels with a previously negative CHV‐1 status to mitigate the risk of disease introduction through latent carriers, thereby safeguarding reproductive health and preventing potential severe consequences for seronegative pregnant bitches (Rota et al. 2020).

Blood specimens were collected from canine populations exhibiting clinical disease manifestations within dog shelters and collection centres located in Konya and Antalya (in Turkey). A total of 141 canine blood samples were subjected to commercial ELISA to detect anti‐CHV‐1 antibodies. Concurrently, leukocyte samples derived from the same blood specimens were analysed via immunofluorescence test (IFT) for the isolation of CHV‐1. Madin–Darby canine kidney (MDCK) cell cultures were employed as a host system for virus isolation. Serological analysis revealed a 68.8% prevalence of antibodies against CHV‐1 among the 141 samples tested. Conversely, virus isolation attempts using an IFT on blood leukocytes yielded no positive results. The high seroprevalence rate observed in this study suggests a substantial level of CHV‐1 exposure within the canine population of Konya and Antalya shelters. To mitigate the potential spread of the virus and associated disease, identification and isolation of infected individuals are crucial for effective disease management (Yapici et al. 2018).

A comparative analysis of seroprevalence was conducted using a commercial ELISA and VNT on blood samples collected from dogs housed in Konya and Antalya shelters. The ELISA kit employed utilized intact viral particles as the immunogenic component. Given the limited cytopathic effect induced by CHV‐1, a plaque reduction neutralization test coupled with immunoperoxidase staining was employed to enhance the detection of viral plaques. Neutralizing antibodies were identified in a randomly selected subset of twenty samples.

The ELISA‐based serological analysis revealed a high prevalence of CHV‐1 antibodies, with 87% of the study population testing positive. The ELISA, despite its high sensitivity, is not entirely reliable for confirming CHV‐1 infection due to its low positive predictive value. Therefore, a confirmatory test like the serum neutralization (SN) test is crucial for accurate diagnosis. However, a negative ELISA result is a strong indicator of the absence of CHV‐1 infection. This information is essential for effective disease management and control strategies in canine populations.

A statistically significant positive correlation was observed between ELISA and SN titre values, as determined with a 95% confidence interval. No statistically significant associations were identified between clinical presentation factors and the outcome variable. The high seroprevalence rates observed in the Mexican canine population suggest a prevalent state of latent herpesvirus infection (Lara et al. 2016) (Chen et al. 2021).

### 6.1 Impact of Diagnostic Methodology

The transient and weak humoral immune response elicited by CHV‐1 infection (Ledbetter et al. 2012) means serological assays (SNT, ELISA and IFA) are highly dependent on the timing of sample collection. Studies conducted in a single time point likely significantly underestimate true exposure rates (Gadsden et al. 2012). Furthermore, the lack of standardized antigens and protocols across laboratories impedes direct comparison between studies (Ronsse et al. 2002; Ledbetter et al. 2009b). Molecular methods like PCR offer superior sensitivity for detecting active infection and viral shedding (Schulze and Baumgärtner 1998) (Nöthling et al. 2008), but they fail to identify latently infected, non‐shedding animals. This diagnostic dichotomy explains why studies like Pratelli et al. (2014) found seropositivity but no viral DNA in swabs, whereas others like Losurdo et al. (Ronsse et al. 2002) detected shedding via PCR in clinically affected animals (Losurdo et al. 2018).

### 6.2 Population Factors

Higher prevalence rates are consistently reported in high‐density environments such as breeding kennels and shelters compared to individually owned pets (Krogenæs et al. 2014; Ronsse et al. 2002; Yeşilbağ et al. 2012). This is logically attributed to increased contact rates and stress, a known trigger for viral reactivation (Ledbetter et al. 2009c; Yeşilbağ et al. 2012; Ledbetter 2013). Although some studies report a correlation between age and seroprevalence of CHV‐1 (Gucht and Pensaert 2001), suggesting cumulative exposure, the confounding effect of waning antibodies makes this relationship complex.

### 6.3 The Reproductive Prevalence Paradox

A critical analysis reveals a perplexing disconnect: Although CHV‐1 is an established cause of reproductive failure, several well‐designed studies have failed to find a statistically significant correlation between seropositivity and abortion rates or other reproductive disorders (Krogenæs et al. 2014; Pratelli et al. 2014; Ström Holst et al. 2012). This could be due to the fact that the presence of antibodies indicates prior exposure and likely immunity, whereas reproductive failure is caused by primary infection or reactivation during pregnancy in susceptible (seronegative) dams. This underscores the limitation of cross‐sectional serological studies in assessing cause‐and‐effect for reproductive outcomes. The study by Rota et al. (Krogenæs et al. 2012) supports this, demonstrating that endemicity in a kennel leads to high antibody titres in adults and protection for puppies, whereas the introduction of the virus into a naïve kennel is where the most severe reproductive consequences occur (Rota et al. 2020) (Table 1).

**TABLE 1:** Summary of selected canine herpesvirus‐1 (CHV‐1) seroprevalence studies highlighting methodological heterogeneity.

References	Country	Population	Diagnostic method	Seroprevalence (%)	Key note
Krogenæs et al. (2014)	Norway	Breeding bitches	IPMA	85.5	High‐density population
Dahlbom et al. (2009)	Finland	Dogs	ELISA	81.5	Association with repro issues
Ronsse et al. (2002)	Belgium	Dogs	SNT	45.75	No link to repro failure
Yeşilbağ et al. (2012)	Turkey	Shelter/Dogs	ELISA/VNT	39.3/29.4	Methodological difference
Babaei et al. (2010)	Iran	Dogs	IFA	20.7	
Pratelli et al. (2014)	Italy	Dogs	SNT/IF	14.6/18.6	No viral DNA detected

Abbreviations: ELISA, enzyme‐linked immunosorbent assay; IFA, immunofluorescence antibody; IPMA, immunoperoxidase monolayer assay; SNT, serum neutralization test; VNT, virus neutralization test.

## 7 Conclusion

CHV‐1 remains a globally distributed pathogen of significant concern for canine reproductive and ocular health. This review highlights that the greatest challenge in understanding its true impact lies in the methodological heterogeneity and limitations of existing studies. The virus's weak immunogenicity and latent nature render serological studies inadequate for determining active infection status, whereas PCR‐based studies only capture moments of active shedding (Carmichael 2005).

CHV‐1 is a virus that affects neonatal and adult dogs, causing severe disease and reproductive issues. It is primarily transmitted through birth but can also spread venereally and transplacentally. CHV‐1 establishes a latent carrier state in the body and can reactivate due to stress or immunosuppression. In adults, it can cause ocular symptoms like inflammation and corneal conditions. Variations in seroprevalence rates are seen in different regions, with maternal antibodies protecting puppies. Effective monitoring and management of CHV‐1 infections are crucial to prevent disease transmission and complications in canine populations (Kawakami et al. 2010).

CHV‐1 is globally distributed, with seroprevalence rates ranging from 20% to over 80% in dog populations, depending on region and population density. High‐risk environments include breeding kennels, shelters and multi‐dog households due to stress‐driven viral reactivation and transmission via bodily fluids (e.g., nasal secretions, genital excretions). Asymptomatic carriers are common, perpetuating silent spread (Ledbetter et al. 2006; Ronsse et al. 2002; Decaro et al. 2008).

CHV‐1 diagnosis is done using methods like PCR and histopathology. Existing studies highlight the importance of PCR‐based diagnostics during active infection and caution against overreliance on serology. ELISA and virus neutralization identify antibodies but cannot distinguish active infection from prior exposure. Cross‐reactivity with other herpesviruses (e.g., feline herpesvirus‐1) and poor sensitivity during latency are the limitation of this disease diagnosis (Krogenæs et al. 2012).

There are three methods to treat this disease: supportive care: critical for neonates (e.g., warmth, fluids and antivirals like acyclovir), though survival rates remain low, antivirals: limited efficacy due to poor bioavailability in puppies and rapid disease progression and vaccines: only one modified‐live vaccine (Eurifel CHV) is available in Europe, but its use is restricted to pregnant females and lacks broad validation (Poulet et al. 2001).

Regarding the genetic and antigenic similarity between CHV and FHV‐1, immunoprecipitation studies using antisera to FHV‐1 and CHV revealed that both share virion glycoprotein antigens with apparent molecular weights of approximately 60 and 68 kDa. Two non‐glycosylated, virion‐associated antigens of each virus also displayed weaker cross‐reactivity. Southern blot hybridization experiments indicated that restriction fragments which represented approximately 51% of the FHV‐1 genome hybridized to CHV DNA under conditions which allowed less than 7% base pair mismatch (Rota and Maes 1990). Using VNTs, enzyme‐linked immunosorbent and indirect immunofluorescence assays and immunoblotting analysis, reciprocal cross‐reactivities to the heterologous virus were observed with some polyvalent and monoclonal antibodies. One monoclonal antibody against CHV neutralized FHV‐1 infectivity, and one monoclonal antibody against FHV‐1 inhibited the haemagglutination activity of CHV as well as FHV‐1‐infected mouse serum. The major cross‐reacting proteins were identified as the 143/108 and 60 kDa glycoproteins of FHV‐1 and the 145/112 and 41 kDa glycoproteins of CHV (Limcumpao et al. 1990).

Critical analysis reveals that the link between CHV‐1 and reproductive failure is most severe in naïve populations, whereas endemic infection in kennels may confer protective immunity—a paradox that explains much of the conflicting data in the literature. The ocular manifestations, while increasingly recognized, require careful differential diagnosis from more common ocular pathogens (Ledbetter et al. 2009c).

There are limitations to this disease that require further research in this field (Rezaei et al. 2020; Bautista et al. 2024; Cunningham et al. 2017):

– **Vaccine development**: No universally approved vaccine exists; strategies targeting latency or mucosal immunity are underexplored. Future research should prioritize longitudinal studies to clarify latency mechanisms and vaccine trials targeting ocular mucosal immunity, and we need more broadly available and effective immunization strategies, particularly for seronegative dams entering high‐risk environments.
– **Diagnostic refinement and standardizing diagnostic protocols**: Improved assays to differentiate latent versus active infection and point‐of‐care tools for rapid outbreak management. Developing and validating universally accepted serological and molecular assays is paramount for accurate prevalence mapping.
– **Pathogenesis**: Mechanisms of immune evasion, latency/reactivation triggers and viral persistence in tissues (e.g., reproductive tract) remain poorly understood.
– **Therapeutics**: Need for CHV‐1‐specific antivirals with better neonatal efficacy and studies on immunomodulators to mitigate reactivation.
– **Epidemiology**: Sparse data on CHV‐1 prevalence in stray/feral dogs and its role in wild canid populations.
– **One Health implications**: Potential zoonotic risks (though none confirmed) and interactions with other herpesviruses warrant investigation.
– **Longitudinal studies**: Tracking dogs over time, through pregnancy and stress events, is needed to truly understand latency, reactivation dynamics and the protective role of antibodies.
– **Addressing diagnostic gaps**: Research into point‐of‐care tests and methods to distinguish latent from active infection would be a clinical breakthrough.

In conclusion, moving beyond mere literature compilation to a critical appraisal of evidence reveals that effective management of CHV‐1 hinges on improved diagnostic clarity and a nuanced understanding of its epidemiology, rather than just its presence or absence in a population.

## Author Contributions

**Mohammadreza Ghorani**: conceptualization, writing – review and editing. **Amir Mohammad Naghshe Javaheri**: writing – original draft. **Mahdiye Shirafkan**: writing – original draft. **Sina Soleimani**: editing. **Hadi Bakhtiari**: conceptualization.

## Funding

The authors have nothing to report.

## Ethics Statement

The authors confirm that the ethical policies of the journal, as noted on the journal's author guidelines page, have been adhered to. No ethical approval was required as this is a review article with no original research data.

## Conflicts of Interest

The authors declare no conflicts of interest.

## Data Availability

The data supporting this study's findings are available from the corresponding author upon reasonable request.

## References

[vms370682-bib-0001] Anvik, J . “Clinical Considerations of Canine Herpesvirus Infection.” 1991. Veterinary Medicine 86, no. 4: 394–403.

[vms370682-bib-0002] Appel, M. , M. Menegus , and I. Parsomon . 1969. “Pathogenesis of Canine Herpesvirus in Specific‐Pathogen‐Free Dogs: 5‐ to 12‐Week‐Old Pups.” American Journal of Veterinary Research 30, no. 12: 2067–2073. 4311021

[vms370682-bib-0003] Babaei, H. , B. Akhtardanesh , R. Ghanbarpour , and A. Namjoo . 2010. “Serological Evidence of Canine Herpesvirus‐1 in Dogs of Kerman City, South‐East of Iran.” Transboundary and Emerging Diseases 57, no. 5: 348–351. 20642493 10.1111/j.1865-1682.2010.01155.x

[vms370682-bib-0004] Bautista, L. , C. Sirimanotham , J. Espinoza , D. Cheng , S. Tay , and N. Drayman . 2024. “A Drug Repurposing Screen Identifies Decitabine as an HSV‐1 Antiviral.” Microbiology Spectrum 12, no. 11: e01754. 24. 39287456 10.1128/spectrum.01754-24 PMC11537057

[vms370682-bib-0005] Bottinelli, M. , E. Rampacci , V. Stefanetti , et al. 2016. “Serological and Biomolecular Survey on Canine Herpesvirus‐1 Infection in a Dog Breeding Kennel.” Journal of Veterinary Medical Science 78, no. 5: 797–802. 26726105 10.1292/jvms.15-0543 PMC4905834

[vms370682-bib-0006] Bujko, M. , V. Sulović , V. Zivanović , B. Lako , and R. Dotlić . 1988. “Effect of Progesterone and Pregnancy on the Replication of Herpes Simplex Virus Type 2 In Vivo.” Clinical and Experimental Obstetrics & Gynecology 15, no. 1–2: 34–37. 2834118

[vms370682-bib-0007] Buonavoglia, C. , V. Martella , A. Pratelli , et al. 2001. “Evidence for Evolution of Canine Parvovirus Type 2 in Italy.” Journal of General Virology 82, no. 12: 3021–3025. 11714979 10.1099/0022-1317-82-12-3021

[vms370682-bib-0008] Burr, P. , M. Campbell , L. Nicolson , and D. Onions . 1996. “Detection of Canine Herpesvirus 1 in a Wide Range of Tissues Using the Polymerase Chain Reaction.” Veterinary Microbiology 53, no. 3–4: 227–237. 9008334 10.1016/s0378-1135(96)01227-8

[vms370682-bib-0009] Cargnelutti, J. F. , E. K. Masuda , M. G. Neuls , R. Weiblen , and E. F. Flores . 2015. “Outbreaks of Canid Herpesvirus 1 Disease in Puppies in Southern Brazil.” Pesquisa Veterinária Brasileira 35: 557–561.

[vms370682-bib-0010] Carmichael, L. , R. Squire , and L. Krook . 1965. “Clinical and Pathologic Features of a Fatal Viral Disease of Newborn Pups.” American Journal of Veterinary Research 26, no. 113: 803–814. 5892835

[vms370682-bib-0011] Carmichael, L. E. 2005. “An Annotated Historical Account of Canine Parvovirus.” Journal of Veterinary Medicine B, Infectious Diseases and Veterinary Public Health 52, no. 7–8: 303–311. 16316389 10.1111/j.1439-0450.2005.00868.x

[vms370682-bib-0012] Chen, Y. , J. Wang , Z. Bi , et al. 2021. “Molecular Epidemiology and Genetic Evolution of Canine Parvovirus in East China, During 2018–2020.” Infection, Genetics and Evolution: Journal of Molecular Epidemiology and Evolutionary Genetics in Infectious Diseases 90: 104780. 33639306 10.1016/j.meegid.2021.104780

[vms370682-bib-0013] Cunningham, A. A. , P. Daszak , and J. L. Wood . 2017. “One Health, Emerging Infectious Diseases and Wildlife: Two Decades of Progress?” Philosophical Transactions of the Royal Society B: Biological Sciences 372, no. 1725: 20160167. 10.1098/rstb.2016.0167 PMC5468692 28584175

[vms370682-bib-0014] Dahlbom, M. , M. Johnsson , V. Myllys , J. Taponen , and M. Andersson . 2009. “Seroprevalence of Canine Herpesvirus‐1 and *Brucella canis* in Finnish Breeding Kennels With and Without Reproductive Problems.” Reproduction in Domestic Animals 44, no. 1: 128–131. 18992103 10.1111/j.1439-0531.2007.01008.x

[vms370682-bib-0015] Dahlbom, M. , M. Johnsson , V. Myllys , J. Taponen , and M. Andersson . 2009. “Seroprevalence of Canine Herpesvirus‐1 and *Brucella canis* in Finnish Breeding Kennels With and Without Reproductive Problems.” Reproduction in Domestic Animals 44, no. 1: 128–131. 18992103 10.1111/j.1439-0531.2007.01008.x

[vms370682-bib-0016] Decaro, N. , F. Amorisco , C. Desario , et al. 2010. “Development and Validation of a Real‐Time PCR Assay for Specific and Sensitive Detection of Canid Herpesvirus 1.” Journal of Virological Methods 169, no. 1: 176–180. 20674611 10.1016/j.jviromet.2010.07.021 PMC7112867

[vms370682-bib-0017] Decaro, N. , V. Martella , and C. Buonavoglia . 2008. “Canine Adenoviruses and Herpesvirus.” Veterinary Clinics of North America: Small Animal Practice 38, no. 4: 799–814. 18501279 10.1016/j.cvsm.2008.02.006 PMC7114865

[vms370682-bib-0018] Evermann, J. 1989. Diagnosis of Canine Herpetic Infections. Current Veterinary Therapy X Philadelphia. WB Saunders.

[vms370682-bib-0019] Evermann, J. F. , E. C. Ledbetter , and R. K. Maes . 2011. “Canine Reproductive, Respiratory, and Ocular Diseases Due to Canine Herpesvirus.” Veterinary Clinics of North America Small Animal Practice 41, no. 6: 1097–1120. 22041206 10.1016/j.cvsm.2011.08.007 PMC7114841

[vms370682-bib-0020] Gadsden, B. J. , R. K. Maes , A. G. Wise , M. Kiupel , and I. M. Langohr . 2012. “Fatal Canid Herpesvirus 1 Infection in an Adult Dog.” Journal of Veterinary Diagnostic Investigation: Official Publication of the American Association of Veterinary Laboratory Diagnosticians Inc 24, no. 3: 604–607. 22529135 10.1177/1040638712440994

[vms370682-bib-0021] Gervais, K. J. , C. G. Pirie , E. C. Ledbetter , and S. Pizzirani . 2012. “Acute Primary Canine Herpesvirus‐1 Dendritic Ulcerative Keratitis in an Adult Dog.” Veterinary Ophthalmology 15, no. 2: 133–138. 22051326 10.1111/j.1463-5224.2011.00952.x

[vms370682-bib-0022] Greene, C. E. 2005. Greene's Infectious Diseases of the Dog and Cat. Elsevier.

[vms370682-bib-0023] Greene, C. E. , and L. E. Carmichael . 2006. “Canine Herpesvirus Infection.” Infectious Diseases of the Dog and Cat 4: 48–54.

[vms370682-bib-0024] Gucht, S. , and M. Pensaert . 2001. “Prevalence of Canine Herpesvirus in Kennels and the Possible Association With Fertility Problems and Neonatal Death.” Vlaams Diergeneeskundig Tijdschrift 70: 204–211.

[vms370682-bib-0025] Gucht, S. V. , H. Nauwynck , and M. Pensaert . 2001. “Prevalence of Canine Herpesvirus in Kennels and the Possible Association With Fertility Problems and Neonatal Death.” Vlaams Diergeneeskundig Tijdschrift 70: 204–211.

[vms370682-bib-0026] Hashimoto, A. , K. Hirai , K. Okada , and Y. Fujimoto . 1979. “Pathology of the Placenta and Newborn Pups With Suspected Intrauterine Infection of Canine Herpesvirus.” American Journal of Veterinary Research 40, no. 9: 1236–1240. 230772

[vms370682-bib-0027] Hashimoto, A. , K. Hirai , T. Yamaguchi , and Y. Fujimoto . 1982. “Experimental Transplacental Infection of Pregnant Dogs With Canine Herpesvirus.” American Journal of Veterinary Research 43, no. 5: 844–850. 6283965

[vms370682-bib-0028] Il‐bok isni, S. , S. Whan‐woo , and L. Chang‐hyeong . 1994. “Survey on the Seroepidemiology of Canine Herpesvirus Infection in Korea.” Korean Journal of Veterinary Research 34, no. 3: 647–652.

[vms370682-bib-0029] Kawakami, K. , H. Ogawa , K. Maeda , et al. 2010. “Nosocomial Outbreak of Serious Canine Infectious Tracheobronchitis (Kennel Cough) Caused by Canine Herpesvirus Infection.” Journal of Clinical Microbiology 48, no. 4: 1176–1181. 20107103 10.1128/JCM.02128-09 PMC2849577

[vms370682-bib-0030] Kraft, S. , J. Evermann , A. McKeirnan , and M. Riggs . 1986. “The Role of Neonatal Canine Herpesvirus Infection in Mixed Infections in Older Dogs.” Compendium on Continuing Education for the Practicing Veterinarian 8: 688–696.

[vms370682-bib-0031] Krogenæs, A. , V. Rootwelt , S. Larsen , et al. 2012. “A Serologic Study of Canine herpes Virus‐1 Infection in the Norwegian Adult Dog Population.” Theriogenology 78, no. 1: 153–158. 22494683 10.1016/j.theriogenology.2012.01.031

[vms370682-bib-0032] Krogenæs, A. , V. Rootwelt , S. Larsen , L. Renström , W. Farstad , and A. Lund . 2014. “A Serological Study of Canine Herpesvirus‐1 Infection in a Population of Breeding Bitches in Norway.” Acta Veterinaria Scandinavica 56, no. 1: 19. 24694206 10.1186/1751-0147-56-19 PMC4021736

[vms370682-bib-0033] Lacheretz, A. , and S. Cognard . 1998. “Epidemiologie Et Diagnostic Serologique De L'herpevirose Canine.” Revue De Medecine Veterinaire (France) 149, no. 8.

[vms370682-bib-0034] Lara, E. G. V. , S. Jiá , and C. C. Verde , et al. 2016. “Canine Herpesvirus Seroprevalence and Associated Factors in Dogs of Mexico.” Open Journal of Veterinary Medicine 6, no. 10: 149.

[vms370682-bib-0035] Larsen, R. W. , M. Kiupel , H.‐J. Balzer , and J. S. Agerholm . 2015. “Prevalence of Canid Herpesvirus‐1 Infection in Stillborn and Dead Neonatal Puppies in Denmark.” Acta Veterinaria Scandinavica 57: 1–7. 25567292 10.1186/s13028-014-0092-9 PMC4296690

[vms370682-bib-0036] Ledbetter, E. 2013. “Canine Herpesvirus‐1 Ocular Diseases of Mature Dogs.” New Zealand Veterinary Journal 61, no. 4: 193–201. 23438442 10.1080/00480169.2013.768151

[vms370682-bib-0037] Ledbetter, E. C. , E. C. da Silva , S. G. Kim , E. J. Dubovi , and W. S. Schwark . 2012. “Frequency of Spontaneous Canine Herpesvirus‐1 Reactivation and Ocular Viral Shedding in Latently Infected Dogs and Canine Herpesvirus‐1 Reactivation and Ocular Viral Shedding Induced by Topical Administration of Cyclosporine and Systemic Administration of Corticosteroids.” American Journal of Veterinary Research 73, no. 7: 1079–1084. 22738061 10.2460/ajvr.73.7.1079

[vms370682-bib-0038] Ledbetter, E. C. , E. J. Dubovi , S. G. Kim , D. J. Maggs , and R. C. Bicalho . 2009. “Experimental Primary Ocular Canine Herpesvirus‐1 Infection in Adult Dogs.” American Journal of Veterinary Research 70, no. 4: 513–521. 19335108 10.2460/ajvr.70.4.513

[vms370682-bib-0039] Ledbetter, E. C. , W. E. Hornbuckle , and E. J. Dubovi . 2009. “Virologic Survey of Dogs With Naturally Acquired Idiopathic Conjunctivitis.” Journal of the American Veterinary Medical Association 235, no. 8: 954–959. 19827980 10.2460/javma.235.8.954

[vms370682-bib-0040] Ledbetter, E. C. , S. G. Kim , and E. J. Dubovi . 2009b. “Outbreak of Ocular Disease Associated With Naturally‐Acquired Canine Herpesvirus‐1 Infection in a Closed Domestic Dog Colony.” Veterinary Ophthalmology 12, no. 4: 242–247. 19604340 10.1111/j.1463-5224.2009.00709.x

[vms370682-bib-0041] Ledbetter, E. C. , S. G. Kim , E. J. Dubovi , and R. C. Bicalho . 2009c. “Experimental Reactivation of Latent Canine Herpesvirus‐1 and Induction of Recurrent Ocular Disease in Adult Dogs.” Veterinary Microbiology 138, no. 1–2: 98–105. 19345521 10.1016/j.vetmic.2009.03.013

[vms370682-bib-0042] Ledbetter, E. C. , R. C. Riis , T. J. Kern , N. J. Haley , and S. J. Schatzberg . 2006. “Corneal Ulceration Associated With Naturally Occurring Canine Herpesvirus‐1 Infection in Two Adult Dogs.” Journal of the American Veterinary Medical Association 229, no. 3: 376–384. 16881829 10.2460/javma.229.3.376

[vms370682-bib-0043] Leontides, L. , C. Ewald , and P. Willeberg . 1994. “Herd Risk Factors for Serological Evidence of Aujeszky's Disease Virus Infection of Breeding Sows in Northern Germany (1990–1991).” Journal of Veterinary Medicine, Series B 41, no. 1–10: 554–560. 7701869 10.1111/j.1439-0450.1994.tb00263.x

[vms370682-bib-0044] Li, Y. , H. Negussie , Y. Qiu , V. R. Reddy , B. Mateusen , and H. J. Nauwynck . 2016. “Early Events of Canine Herpesvirus 1 Infections in Canine Respiratory and Genital Mucosae by the Use of Ex Vivo Models.” Research in Veterinary Science 105: 205–208. 27033934 10.1016/j.rvsc.2016.02.019

[vms370682-bib-0045] Limcumpao, J. A. , T. Horimoto , X. Xuan , E. Takahashi , and T. Mikami . 1990. “Immunological Relationship Between Feline Herpesvirus Type 1 (FHV‐1) and Canine Herpesvirus (CHV) as Revealed by Polyvalent and Monoclonal Antibodies.” Archives of Virology 111, no. 3–4: 165–176. 2162158 10.1007/BF01311051

[vms370682-bib-0046] Losurdo, M. , G. Dowgier , M. S. Lucente , et al. 2018. “Long‐Term Shedding of Canine Alphaherpesvirus 1 in Naturally Infected Newborn Pups.” Research in Veterinary Science 119: 244–246. 30005400 10.1016/j.rvsc.2018.07.001 PMC7172181

[vms370682-bib-0047] Maes, D. , H. Deluyker , M. Verdonck , et al. 2000. “Herd Factors Associated With the Seroprevalences of Four Major Respiratory Pathogens in Slaughter Pigs From Farrow‐to‐Finish Pig Herds.” Veterinary Research 31, no. 3: 313–327. 10863948 10.1051/vetres:2000122

[vms370682-bib-0048] Malone, E. , E. Ledbetter , K. Rassnick , S. Kim , and D. Russell . 2010. “Disseminated Canine Herpesvirus‐1 Infection in an Immunocompromised Adult Dog.” Journal of Veterinary Internal Medicine 24, no. 4: 965–968. 20412440 10.1111/j.1939-1676.2010.0512.x

[vms370682-bib-0049] McDermott, J. , M. Kadohira , C. O'Callaghan , and M Shoukri . 1997. “A Comparison of Different Models for Assessing Variations in the Sero‐Prevalence of Infectious Bovine Rhinotracheitis by Farm, Area and District in Kenya.” Preventive Veterinary Medicine 32, no. 3–4: 219–234. 9443329 10.1016/s0167-5877(97)00025-1

[vms370682-bib-0050] Miyoshi, M. , Y. Ishii , M. Takiguchi , et al. 1999. “Detection of Canine Herpesvirus DNA in the Ganglionic Neurons and the Lymph Node Lymphocytes of Latently Infected Dogs.” Journal of Veterinary Medical Science 61, no. 4: 375–379. 10342288 10.1292/jvms.61.375

[vms370682-bib-0051] Morresey, P. R. 2004. “Reproductive Effects of Canine Herpesvirus.” Compendium 26, no. 10: 804–810.

[vms370682-bib-0052] Nöthling, J. , D. Hüssy , D. Steckler , and M. Ackermann . 2008. “Seroprevalence of Canine Herpesvirus in Breeding Kennels in the Gauteng Province of South Africa.” Theriogenology 69, no. 3: 276–282. 17981320 10.1016/j.theriogenology.2007.09.022

[vms370682-bib-0054] Okuda, Y. , A. Hashimoto , T. Yamaguchi , et al. 1993. “Repeated Canine Herpesvirus (CHV) Reactivation in Dogs by an Immunosuppressive Drug.” Cornell Veterinarian 83, no. 4: 291–302. 8306652

[vms370682-bib-0055] Okuda, Y. , K. Ishida , A. Hashimoto , et al. 1993. “Virus Reactivation in Bitches With a Medical History of Herpesvirus Infection.” American Journal of Veterinary Research 54, no. 4: 551–554. 8387252

[vms370682-bib-0056] Poulet, H. , P. Guigal , M. Soulier , et al. 2001. “Protection of Puppies Against Canine Herpesvirus by Vaccination of the Dams.” Veterinary Record 148, no. 22: 691–695. 11425256 10.1136/vr.148.22.691

[vms370682-bib-0057] Pratelli, A. , V. Colao , and M. Losurdo . 2014. “Serological and Virological Detection of Canine Herpesvirus‐1 in Adult Dogs With and Without Reproductive Disorders.” Veterinary Journal (London, England: 1997) 200, no. 2: 257–260. 24685471 10.1016/j.tvjl.2014.03.001

[vms370682-bib-0058] Rezaei, M. , M. Jajarmi , R. Alizadeh , M. Khalili , and H. Babaei . 2020. “First Molecular Study of Canine Herpesvirus‐1 in Reproductive Specimens of Adult Dogs in Southeast of Iran.” Comparative Immunology, Microbiology and Infectious Diseases 71: 101487. 32339866 10.1016/j.cimid.2020.101487

[vms370682-bib-0059] Ronsse, V. , J. Verstegen , K. Onclin , et al. 2002. “Seroprevalence of Canine Herpesvirus‐1 in the Belgian Dog Population in 2000.” Reproduction in Domestic Animals=Zuchthygiene 37, no. 5: 299–304. 12354184 10.1046/j.1439-0531.2002.00363.x

[vms370682-bib-0060] Ronsse, V. , J. Verstegen , K. Onclin , F. Farnir , and H. Poulet . 2004. “Risk Factors and Reproductive Disorders Associated With Canine Herpesvirus‐1 (CHV‐1).” Theriogenology 61, no. 4: 619–636. 14698053 10.1016/s0093-691x(03)00249-8

[vms370682-bib-0061] Ronsse, V. , J. Verstegen , E. Thiry , et al. 2005. “Canine Herpesvirus‐1 (CHV‐1): Clinical, Serological and Virological Patterns in Breeding Colonies.” Theriogenology 64, no. 1: 61–74. 15935843 10.1016/j.theriogenology.2004.11.016

[vms370682-bib-0062] Rota, A. , A. Dogliero , T. Biosa , M. Messina , P. Pregel , and L. Masoero . 2020. “Seroprevalence of Canine Herpesvirus‐1 in Breeding Dogs With or Without Vaccination in Northwest Italy.” Animals: An Open Access Journal From MDPI 10, no. 7: 1116. 32610623 10.3390/ani10071116 PMC7401649

[vms370682-bib-0063] Rota, P. A. , and R. K. Maes . 1990. “Homology Between Feline Herpesvirus‐1 and Canine Herpesvirus.” Archives of Virology 115, no. 1–2: 139–145. 2174232 10.1007/BF01310631

[vms370682-bib-0064] Schulze, C. , and W. Baumgärtner . 1998. “Nested Polymerase Chain Reaction and In Situ Hybridization for Diagnosis of Canine Herpesvirus Infection in Puppies.” Veterinary Pathology 35, no. 3: 209–217. 9598584 10.1177/030098589803500306

[vms370682-bib-0066] Spertzel, R. , D. Huxsoll , S. McConnell , L. Binn , and R. Yager . 1965. “Recovery and Characterization of a Herpes‐Like Virus From Dog Kidney Cell Cultures.” Proceedings of the Society for Experimental Biology and Medicine 120, no. 3: 651–655. 5858689 10.3181/00379727-120-30615

[vms370682-bib-0067] Stewart, S. E. , J. David‐Ferreira , E. Lovelace , J. Landon , and N. Stock . 1965. “Herpes‐Like Virus Isolated From Neonatal and Fetal Dogs.” Science 148, no. 3675: 1341–1343. 14281717 10.1126/science.148.3675.1341

[vms370682-bib-0068] Ström Holst, B. , M. Hagberg Gustavsson , M. Grapperon‐Mathis , et al. 2012. “Canine Herpesvirus During Pregnancy and Non‐Pregnant Luteal Phase.” Reproduction in Domestic Animals=Zuchthygiene 47, no. S6: 362–365. 23279539 10.1111/rda.12099

[vms370682-bib-0069] Sykes, J. , and E Greene . 2012. Infectious Diseases of the Dog and Cat. Saunders. Elsevier Inc.

[vms370682-bib-0070] Thiry, E. , J. Saliki , A. Schwers , and P.‐P. Pastoret . 1985. “Parturition as a Stimulus of IBR Virus Reactivation.” Veterinary Record 116, no. 22: 599–600. 2990084 10.1136/vr.116.22.599

[vms370682-bib-0071] Yapici, O. , O. Avci , O. Bulut , et al. 2018. “Detection of Canine Herpesvirus Infection on Dogs.” Microbiology Research Journal International 24, no. 1: 1–6.

[vms370682-bib-0072] Yeşilbağ, K. , E. Yalçın , P. Tuncer , and Z. Yılmaz . 2012. “Seroprevalence of Canine Herpesvirus‐1 in Turkish Dog Population.” Research in Veterinary Science 92, no. 1: 36–39. 21075406 10.1016/j.rvsc.2010.10.016

